# A Microarray-Based Genetic Screen for Yeast Chronological Aging Factors

**DOI:** 10.1371/journal.pgen.1000921

**Published:** 2010-04-22

**Authors:** Mirela Matecic, Daniel L. Smith, Xuewen Pan, Nazif Maqani, Stefan Bekiranov, Jef D. Boeke, Jeffrey S. Smith

**Affiliations:** 1 Department of Biochemistry and Molecular Genetics, University of Virginia Health System, School of Medicine, Charlottesville, Virginia, United States of America; 2 Department of Molecular Biology and Genetics, High Throughput Biology Center, Johns Hopkins University School of Medicine, Baltimore, Maryland, United States of America; Stanford University Medical Center, United States of America

## Abstract

Model organisms have played an important role in the elucidation of multiple genes and cellular processes that regulate aging. In this study we utilized the budding yeast, *Saccharomyces cerevisiae*, in a large-scale screen for genes that function in the regulation of chronological lifespan, which is defined by the number of days that non-dividing cells remain viable. A pooled collection of viable haploid gene deletion mutants, each tagged with unique identifying DNA “bar-code” sequences was chronologically aged in liquid culture. Viable mutants in the aging population were selected at several time points and then detected using a microarray DNA hybridization technique that quantifies abundance of the barcode tags. Multiple short- and long-lived mutants were identified using this approach. Among the confirmed short-lived mutants were those defective for autophagy, indicating a key requirement for the recycling of cellular organelles in longevity. Defects in autophagy also prevented lifespan extension induced by limitation of amino acids in the growth media. Among the confirmed long-lived mutants were those defective in the highly conserved *de novo* purine biosynthesis pathway (the *ADE* genes), which ultimately produces IMP and AMP. Blocking this pathway extended lifespan to the same degree as calorie (glucose) restriction. A recently discovered cell-extrinsic mechanism of chronological aging involving acetic acid secretion and toxicity was suppressed in a long-lived *ade4*Δ mutant and exacerbated by a short-lived *atg16*Δ autophagy mutant. The identification of multiple novel effectors of yeast chronological lifespan will greatly aid in the elucidation of mechanisms that cells and organisms utilize in slowing down the aging process.

## Author Summary

The aging process is associated with the onset of several age-associated diseases including diabetes and cancer. In rodent model systems, the dietary regimen known as caloric restriction (CR) is known to delay or prevent these diseases and to extend lifespan. As a result, there is a great deal of interest in understanding the mechanisms by which CR functions. The budding yeast, *Saccharomyces cerevisiae*, has proven to be an effective model for the analysis of genes and cellular pathways that contribute to the regulation of aging. In this study we have performed a microarray-based genetic screen in yeast that identified short- and long-lived mutants from a population that contained each of the viable haploid gene deletion mutants from the yeast gene knockout collection that were pooled together. Using such an approach, we were able to identify genes from several pathways that had not been previously implicated in aging, including some that appear to contribute to the CR effect induced by restriction of either amino acids or sugar. These results are expected to provide new groundwork for future mechanistic aging studies in more complex organisms.

## Introduction

Model eukaryotic organisms such as *Drosophila* and *C. elegans* have played important roles in the identification of genes and the molecular characterization of cellular and biochemical pathways that affect the aging process [1]. For example, large-scale systematic RNAi knockdown screens for lifespan extension with *C. elegans* have implicated multiple genes that regulate metabolism, signal transduction, protein turnover, and gene expression [2], [3]. The budding yeast, *Saccharomyces cerevisiae*, has also been particularly useful, especially in characterizing the NAD^+^-dependent protein deacetylase, Sir2, as a replicative lifespan (RLS) factor [4]. RLS is defined by the number of mitotic cell divisions that a mother cell undergoes prior to senescencing [5].

Yeast lifespan can also be measured chronologically, where the time that non-dividing cells remain viable is monitored [6]. This chronological lifespan (CLS) is typically measured in cells that have entered stationary phase (G_0_). Both types of yeast aging share multiple effectors of lifespan related to nutrient signaling. Deletion of *SCH9* extends both RLS and CLS [6], [7]. Sch9 is related to the serine/threonine kinase (Akt), that in higher eukaryotes functions in insulin-like growth factor (IGF) signaling pathways that have been linked to lifespan regulation [6]. Mutations in the Target of Rapamycin (TOR) signaling pathway also extend both types of lifespan in yeast [8]–[10], as well as in *C. elegans* [11]. The overlap between CLS and RLS extends to the effects of calorie restriction (CR), a dietary regimen shown to extend the mean and maximum lifespan of rodents [12]. In the yeast system, CR consists of reducing the glucose concentration in the growth medium from the non-restricted (NR) level of 2% (w/v) to the CR level of 0.5% or lower [13], [14]. CR extends both RLS and CLS [13]–[16], consistent with the general theme of conserved nutrient signaling pathways playing major roles in longevity. CR, *sch9*Δ, and *tor1*Δ conditions all cause a shift in glucose metabolism from fermentation toward respiration in both lifespan systems [10], [16], [17], revealing a strong link with mitochondrial function. Despite the numerous similarities in nutrient-mediated responses between RLS and CLS, there are also significant differences. One of the most striking is that while *SIR2* promotes RLS and is reported to be required for lifespan extension by CR [14], deletion of *SIR2* mildly extends CLS and is not required for CR-mediated lifespan extension in this system [15], [16]. Instead, Sir2-mediated deacetylation of the gluconeogenesis enzyme Pck1 limits the large extension of CLS caused by extreme CR conditions [18].

Due to its simplicity, CLS has been amenable to genome-wide functional aging screens. A previous screen for long-lived mutants used the yeast knockout (YKO) collection of individual diploid deletion mutants to individually test each mutant for CLS while incubating in 96-well plates. Several deletion mutants downstream of the TOR signaling pathway were identified, thus implicating TOR signaling in lifespan control [8]. In our study we have utilized the YKO collection to identify additional genetic factors that influence CLS through a different approach. A microarray-based genetic screen was performed on the collection, with the goal of determining which deletion mutants shorten or extend lifespan under NR or CR growth conditions. We report the identification of several classes of short-lived mutants, including those that affect mitochondrial function and the autophagy pathway. We also identify and characterize long-lived mutants in the highly conserved *de novo* purine biosynthesis pathway that generates IMP, AMP, and GMP. Deletion of genes in this pathway extended lifespan equally to the effect of CR, and CR did not further extend the lifespan of the mutants, suggesting that there are overlapping mechanisms between these two conditions that promote longevity. We show that the *de novo* purine biosynthesis mutants alter the surrounding growth medium in a way that extends the lifespan of WT cells, pointing to a cell-extrinsic component of CLS regulation.

## Results

### A microarray-based screen for yeast genes involved in chronological life span

**Figure 1:**
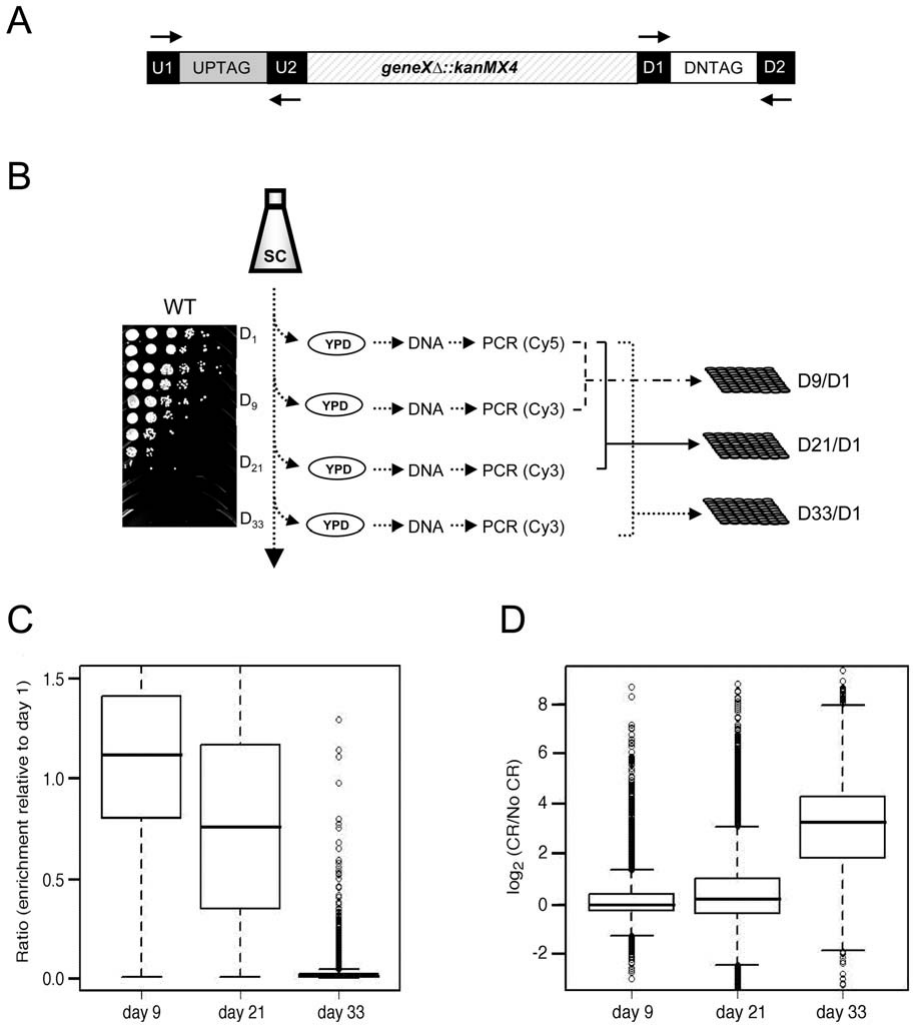
Microarray-based screen for chronological longevity factors. (A) Schematic representation of the UP and DOWN tags flanking *KanMX*. Universal primer sequences (U1/U2 and D1/D2) flank the UP and DOWN tags. (B) Experimental flow of the screen. Aliquots were removed from SC cultures of the pooled YKO population at days 1, 9, 21, and 33, and spread onto YPD plates to allow growth of survivors. A typical BY4741 CLS time course in 2% glucose (NR) is shown. The TAGs were PCR amplified from genomic DNA and fluorescently labeled with either Cy5 (day 1) or Cy3 (days 9, 21, and 33). The day 1 TAGs were co-hybridized with day 9, 21, or 33 TAGs onto the microarray to generate the abundance ratio for each mutant at that particular day (D9/D1, D21/D1 and D33/D1). (C) Box plot of the mutant abundance ratios within the aging population at days 9, 21, and 33 from the NR medium. (D) Box plot showing the general increase in mutant viability within the aging population for CR medium compared to NR medium.

We took advantage of the YKO collection of gene deletion mutants [19], in which each individual gene is replaced by the selection marker (*kanMX4*) and flanked by specific UPTAG and DNTAG sequences (Figure 1A). Viable mutants from the haploid collection were pooled together and grown in synthetic complete (SC) medium that contained either 2% glucose (non-restricted/NR) or 0.5% glucose (calorie restricted/CR). On days 1, 9, 21, and 33, aliquots were removed and spread onto YPD plates to recover mutants that remained viable (Figure 1B). The TAG sequences present in the recovered cells were PCR amplified using universal primers labeled with Cy3 for day 1, or Cy5 for days 9, 21, and 33 (Figure 1B). Following microarray co-hybridizations, the relative abundance of each mutant was determined by the ratio of Cy5 signal (days 9, 21, or 33) to the Cy3 signal (day 1). (see Table S1 for ratios).

Under- or over-representation of a particular mutant's DNA in the aging population was predicted to be indicative of its CLS relative to the other mutants. As expected, the abundance ratios of the TAG signals for most mutants decreased over time in the NR culture (Figure 1C), indicating that most mutants in the population lost viability (aged). By day 33, when the WT strain was completely dead (Figure 1B, spot assay), there were a limited number of viable mutants in the population that could potentially be extremely long-lived (Figure 1C, data shown for the NR population). The viability of most mutants at day 33 was greater in the CR growth condition than in the NR condition (Figure 1D), suggesting that most mutants respond to CR by extending their CLS.

### Specific classes of short-lived and CR–unresponsive mutants

**Figure 2:**
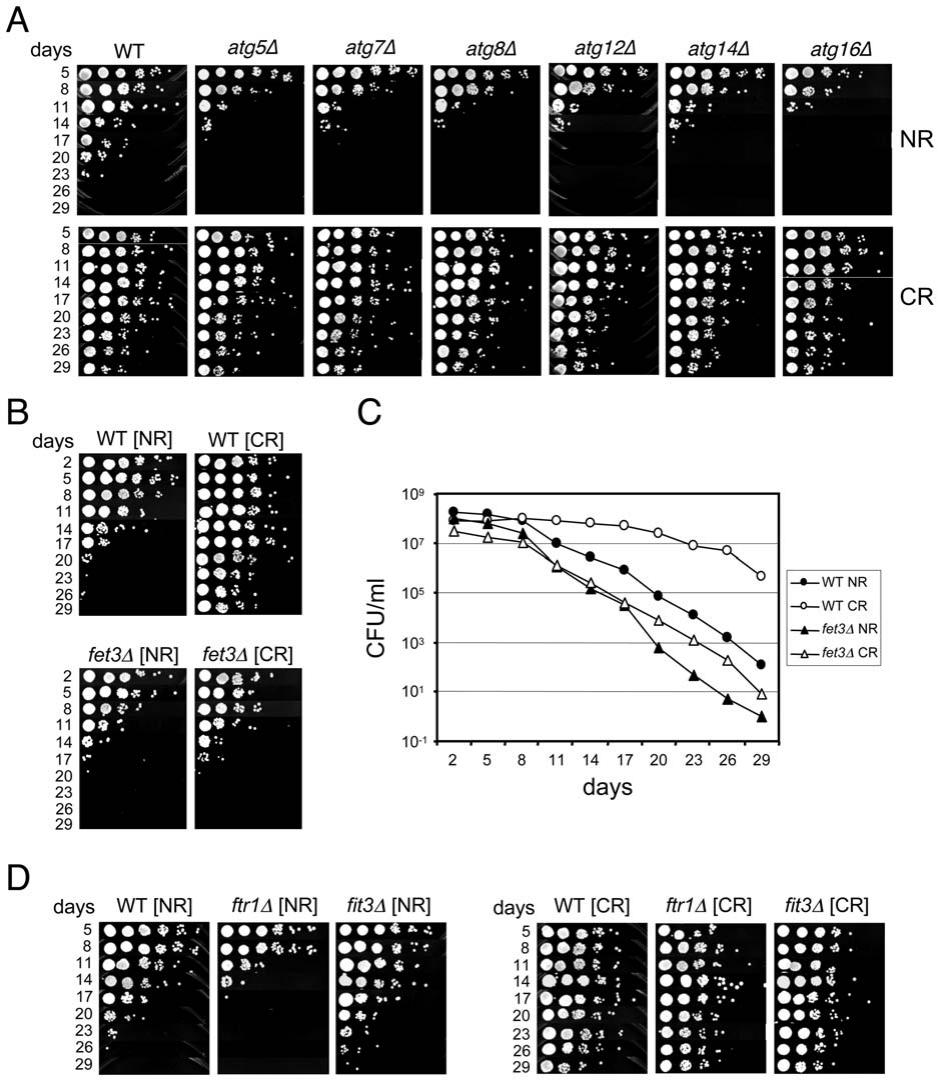
Deletion mutants that shorten CLS. (A) Various deletions of autophagy genes isolated from the screen as short-lived were retested individually for CLS in NR and CR media. (B) Semi-quantitative CLS assay comparing a *fet3*Δ mutant to WT in NR and CR media. (C) Quantitative CLS assay for the same *fet3*Δ and WT strains in NR and CR media. Colony forming units (CFU) are plotted over time. (D) CLS assay showing CR-mediated extension of lifespan in *ftr1*Δ and *fit3*Δ mutants.

To conservatively choose a subset of mutants for retesting the predicted short CLS phenotype, we set two separate threshold criteria. First, the abundance ratios at day 9 for both TAGs had to be ranked in the bottom 200. Second, the abundance ratio at day 21 had to be less than 0.3 for both TAGs, which represented the bottom quartile for this time point (Figure 1C). The day 33 abundance ratios were not considered because most mutants were dead by then (Figure 1C). The result was 117 candidate mutants predicted to be short-lived (Table S2). Out of this list of 117 mutants, we individually retested 16 of them for CLS, and found 13 (81.3%) to actually be short-lived (Table S2). Interestingly, 42 of the 117 candidate genes were related to mitochondrial function in some way (Table S2), most likely because respiration defects prevent cells from properly transitioning through the diauxic shift, thus reducing stationary phase viability [20]. Another major sub-class from the 117 candidates included 10 of the “*ATG*” genes involved in autophagy. As shown in Figure 2A, the autophagy mutants that we directly tested generally caused a short CLS in 2% glucose as predicted by the screen. The CLS of these mutants was fully extended by the CR condition (Figure 2A), which was somewhat surprising because earlier work in *C. elegans* showed that autophagy was required for dietary restriction (DR)-mediated extension of lifespan [21], [22]. All mutants that were tested for various reasons in this study and found to have a short CLS in 2% glucose, including the *atg* mutants, are listed in Table S3.

We were also interested in identifying mutants whose lifespan was not extended by CR. Such mutants were predicted to have similar abundance ratios in the NR and CR conditions across the time course. Many mutants initially appeared to fit this category, which required them to have average NR and CR log rations within 10% of each other (see Materials and Methods). However, only 2 of 41 mutants retested (4.9%) were actually confirmed as being CR-unresponsive. These two affected genes were *NFU1* and *FET3*, both of which encode proteins involved in iron homeostasis. The CLSs of these two mutants were slightly shorter than WT when grown under NR conditions, and, as predicted from the screen, were not extended by CR (Figure 2B and 2C, and data not shown). *NFU1* encodes a mitochondrial matrix protein thought to be involved in iron-sulfur complex biogenesis [23], an important part of the electron transport cascade within the mitochondrial membrane. Its close link with respiration could explain why the *nfu1*Δ mutant had a shorter lifespan in the CR condition than in the NR condition (data not shown).

*FET3* encodes a multicopper oxidase, that along with the iron permease (Ftr1), comprises a high affinity iron uptake system [24], initially suggesting that high affinity transport of iron is required in CR-induced CLS determination. However, even though an *ftr1*Δ mutant exhibited a slight shortening of CLS in the NR condition similar to the *fet3*Δ mutant, CR still induced full CLS extension (Figure 2D). Another protein, Fit3, is one of three secreted mannoproteins that functions in the retention of siderophore-iron in the cell wall, which can be released and then imported by the Fet3/Ftr1 transport system [25]. Deletion of *FIT3* had no affect on CLS, and like the *ftr1*Δ mutant, its CLS was extended by CR (Figure 2D). Taken together, these results suggest that Fet3 may have a function independent of Ftr1-mediated iron transport at the plasma membrane that is important for CLS during CR growth conditions.

### Identification of long-lived mutants

**Figure 3:**
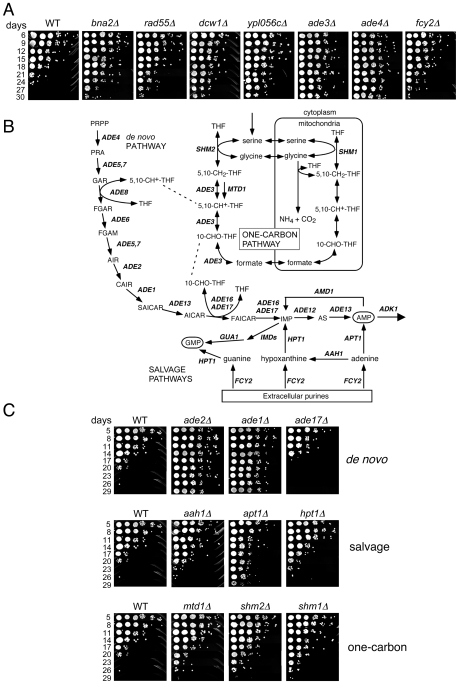
Lifespan-extending mutants include those that block *de novo* and salvage biosynthesis of purines. (A) Examples of CLS assays for various long-lived mutants isolated from the screen, including genes related to purine metabolism (*ade3*Δ, *ade4*Δ, and *fcy2*Δ). (B) Schematic diagram of the *de novo* purine biosynthesis pathway and its connections to one-carbon metabolism and purine import/salvage pathways. Partially adapted from [71]. (C) Examples of additional deletion mutants from the *de novo* purine biosynthesis, purine salvage, and one-carbon metabolism pathways that were not originally isolated from the screen.

To identify long-lived mutants, we again defined conservative thresholds in which the day 33/day1 signal ratio had to be in the top 500 for both the UP-and DN-tags. The day 21/day1 ratio also had to be greater than 1.0 for both TAGs, resulting in a list of 40 mutants (Table 1). Twelve out of the 39 mutants retested (30.7%) had a long CLS (several shown in Figure 3A). Isolation of the *de novo* NAD^+^ biosynthesis gene, *BNA2*, was consistent with the long CLS of a strain lacking *BNA1* [16]. *YPL056C*, *YLR104W*, and *YGL085C*, were previously uncharacterized and have now been named based on their Long Chronological Lifespan phenotype as *LCL1*, *LCL2*, and *LCL3*, respectively. The *lcl1*Δ mutant was previously shown to be resistant to the antifungal drug fluconazole [26], and the *lcl2*Δ mutant has deficient levels of mannosylphosphate in the cell wall [27], suggesting that both of these genes may function in cell wall integrity. *DCW1* encodes a putative mannosidase involved in cell well biosynthesis [28], again pointing to the importance of cell wall structure and function in longevity. *LCL3* encodes a protein with homology to *Staphylococcus aureus* nuclease [29]. Three of the long-lived mutants were involved in either *de novo* purine biosynthesis (*ADE3* and *ADE4*) or purine import (*FCY2*) [30]–[32]. Ade4 catalyzes the first step of the pathway, while Ade3 functions in one-carbon metabolism, which donates tetrahydrofolate-linked carbon units for synthesis of the purine ring (see Figure 3B). Fcy2 is a purine/cytosine permease that mediates transport of purine bases (adenine, guanine, hypoxanthine), and a specific pyrimidine base (cytosine) across the plasma membrane into the cell. Additional mutants were analyzed for CLS outside of the selection criteria. Those mutants that exhibited an extended lifespan under NR conditions are listed in Table S4, while those with a normal lifespan under NR conditions are listed in Table S5.

**Table 1:** Long-lived mutant candidates chosen from screen.

		UPTAG	UPTAG	DNTAG	DNTAG	
		day 33	day 21	day 33	day 21	mutant
ORF	Gene	rank	ratio	rank	ratio	lifespan^a^
*YER056C*	*FCY2*	3	6.91	7	5.47	L
*YPL056C*	***LCL1***	5	3.23	12	3.80	**L**
*YKL046C*	*DCW1*	6	5.06	2	7.84	**L**
*YNL099C*	*OCA1*	8	9.39	10	9.56	N
*YOL013C*	*HRD1*	9	48.76	1	167.92	S
*YBL053W*	*YBL053W*	11	1.41	11	1.35	N
*YJL201W*	*ECM25*	12	1.02	16	1.06	N
*YDR425W*	*SNX41*	16	16.06	13	17.37	**L**
*YCR011C*	*ADP1*	18	26.85	4	42.99	S
*YLL013C*	*PUF3*	22	1.93	5	3.99	S
*YLR154C*	*RNH203*	23	1.66	6	2.04	N
*YLR104W*	***LCL2***	24	6.67	14	20.63	**L**
*YBL052C*	*SAS3*	25	2.27	18	1.71	N
*YPL015C*	*HST2*	39	1.40	20	1.99	N
*YGR204W*	*ADE3*	43	1.21	194	1.10	L
*YJL129C*	*TRK1*	55	9.26	264	9.03	N
*YMR169C*	*ALD3*	75	1.88	51	1.08	S
*YBR057C*	*MUM2*	89	3.71	139	1.47	**L**
*YDR181C*	*SAS4*	94	1.51	481	1.41	**L**
*YFR032C-A*	*RPL29*	129	1.07	501	1.09	N
*YDL118W*	*YDL118W*	135	1.19	218	1.47	N
*YFR008W*	*FAR7*	155	2.37	128	2.27	S
*YOR295W*	*UAF30*	176	2.53	80	1.34	**L**
*YGL003C*	*CDH1*	179	1.40	433	1.36	N
*YGL056C*	*SDS23*	181	1.42	463	1.63	**L**
*YDR485C*	*VPS72*	198	10.70	415	10.22	N
*YGL085W*	***LCL3***	222	1.42	378	1.12	**L**
*YLR043C*	*TRX1*	263	6.95	84	3.03	S
*YER054C*	*GIP2*	285	1.38	324	1.63	N
*YDL122W*	*UBP1*	301	11.51	198	12.43	UN
*YNL144C*	*YNL144C*	309	6.70	468	2.22	N
*YDL059C*	*RAD59*	325	1.59	267	1.57	N
*YDR220C*	*YDR220C*	370	2.35	65	1.75	N
*YLR236C*	*YLR236C*	372	1.68	487	2.03	S
*YBL106C*	*SRO77*	375	3.71	94	3.69	S
*YGR021W*	*YGR021W*	393	1.04	228	2.02	N
*YMR300C*	*ADE4*	438	2.41	83	2.46	L
*YLR207W*	*HRD3*	449	31.26	261	77.13	S
*YGR017W*	*YGR017W*	456	1.81	414	1.91	N
*YDR312W*	*SSF2*	474	1.27	355	1.67	N

**a** Confirmed lifespan phenotype: L = long, S = short, N = normal, UN = untested.

### Characterization of the *ADE* pathway in CLS regulation

The effects of the *de novo* purine biosynthesis pathway on aging have not been well studied. In *Drosophila melanogaster*, mutations in the pathway cause pleiotropic effects due to general purine deficiency, one of them being a short lifespan [33]. In yeast, the pathway was not previously implicated in lifespan regulation. The *de novo* purine biosynthesis pathway is highly conserved and consists of ten consecutive reactions catalyzed by the *ADE* gene products that convert 5-phosphoribosyl 1-pyrophosphate (PRPP) to inosine monophosphate (IMP), which is then used for AMP and GMP synthesis (Figure 3B). There are also purine salvage pathways that either import extracellular purines via Fcy2 or utilize endogenous purines to synthesize IMP, GMP or AMP through only a few enzymatic steps (Figure 3C; for review see [34]). Deleting other genes in the *de novo* synthesis pathway such as *ADE1*, *ADE2*, *ADE5,7*, *ADE6*, or *ADE12* significantly extended CLS (Figure 3C and data not shown). *ADE13* is essential and *ade8*Δ was not available in our KO collection, so they were not tested. The lone exception encountered was an *ade17*Δ mutant, which had a lifespan modestly, but reproducibly, shorter than WT (Figure 3C). Ade17, as well as Ade16, catalyzes the conversion of 5-aminoimidazole-4-carboxamide-1-β-D-ribofuranoside (AICAR) into 5′-phosphoribosyl-5-formaminoimidazole-4-carboxamide (FAICAR). The major enzyme in this step is Ade17, being responsible for ∼90% of AICAR transformylase activity [35]. Mutants in the purine salvage pathways (*AAH1*, *APT1*, or *HPT1*) or the one-carbon metabolism pathway (*MTD1*, *SHM2*, or *SHM1*) also extended CLS, but to a lesser extent than mutants in the *de novo* pathway (Figure 3C). The effects of these two pathways on CLS may, therefore, be mediated by a secondary effect on regulation of the *de novo* pathway.

**Figure 4:**
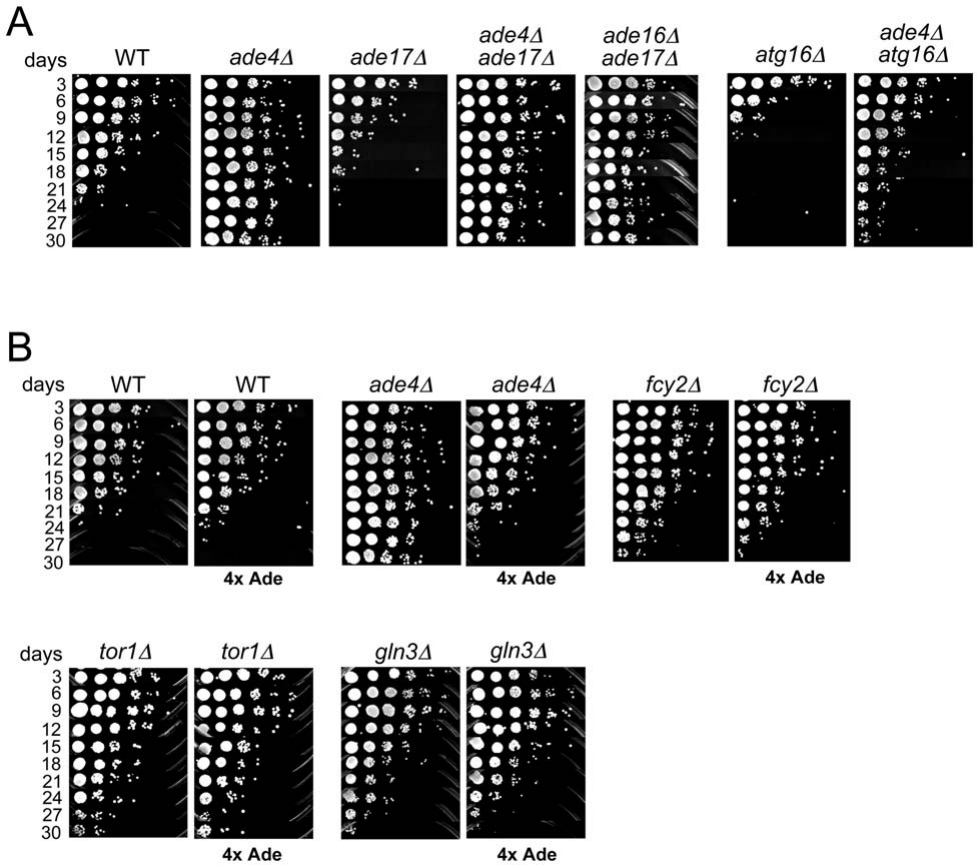
Epistasis analysis of the *de novo* purine biosynthesis pathway in CLS. (A) The lifespan extending *ade4*Δ mutation was combined with lifespan shortening *ade17*Δ and *atg16*Δ mutations through genetic crosses, and the double mutants tested for CLS when grown in SC 2% glucose (NR) medium. An *ade16*Δ *ade17*Δ double mutant that completely blocks the AICAR to FAICAR step of the *de novo* pathway was also tested. (B) CLS assays with WT, *ade4*Δ, *fcy2*Δ, *tor1*Δ, and *gln3*Δ strains grown in standard SC medium that contains (30 mg/L adenine), or SC medium supplemented with 4 times more adenine (4x Ade; 120 mg/L).

The *de novo* synthesis of purine nucleotides is regulated at the genetic and enzymatic levels. Enzymatically, the first step of the pathway catalyzed by Ade4 is feedback-inhibited by the end products ADP and ATP [36]. Genetically, excess adenine has a repressing effect on *ADE* regulon genes, while depletion of adenine results in transcriptional up-regulation due to the activity of transcription factors Bas1 and Bas2/Pho2 [37], [38]. Regulation of all the *de novo* pathway genes, with the exception of *ADE16*, is achieved via the Bas1/Pho2 complex [39]. It is proposed that the AICAR or SAICAR intermediates promote Bas1-Pho2 dimerization, resulting in the up-regulation of *ADE-*gene transcription [36], [38], [40]. Since we observed CLS extension in *ade* mutants lacking an enzyme upstream of the AICAR intermediate and CLS shortening for the *ade17*Δ mutant that likely accumulates AICAR [40], we generated an *ade4*Δ *ade17*Δ double mutant and tested CLS. As shown in Figure 4A, the *ade4*Δ mutation was epistatic to the *ade17*Δ mutation for lifespan in the double mutant, initially consistent with a hypothesis that accumulation of AICAR shortens CLS of the *ade17*Δ mutant. However, completely blocking the AICAR to FAICAR step of the *de novo* pathway with an *ade16*Δ *ade17*Δ double mutant, surprisingly resulted in CLS extension (Figure 4A).

Since excess adenine represses the *de novo* purine synthesis pathway, we next tested whether excess adenine would extend CLS. The SC medium contained either our standard limiting concentration of adenine (30 mg/L) or a 4-fold excess (120 mg/L), which represses the *de novo* pathway. Surprisingly, excess adenine did not extend the CLS of a WT strain, but instead suppressed the long CLS phenotype of *ade2*Δ, *ade3*Δ, or *ade4*Δ mutants (Figure 4B and data not shown). This effect was specific to the long-lived *ade* mutants, because excess adenine did not shorten the CLS of two long-lived mutants with inhibited TOR signaling, *tor1*Δ and *gln3*Δ (Figure 4B). The *fcy2*Δ mutation blocks adenine transport, so the addition of excess adenine did not affect CLS.

**Figure 5:**
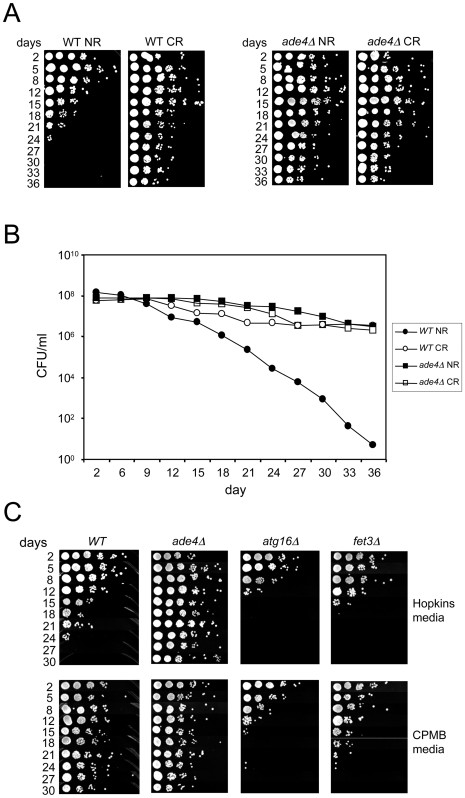
Growth media effects on CLS. (A) Semi-quantitative CLS assay showing that deleting *ADE4* extends CLS to the same degree as CR extends lifespan of a WT strain. The *ade4*Δ mutation and CR are not additive for CLS extension. (B) Quantitative CLS assay showing similarities between lifespan extension caused by the *ade4*Δ mutation and the CR growth condition. CFU  =  colony forming units. (C) SC medium with generally lower concentrations of amino acids (CPMB media) extends CLS when compared to the richer SC medium used in the genetic screen (Hopkins media). Deleting *ATG16* (blocking autophagy) or *FET3* (iron metabolism) prevented the extension of CLS induced by CPMB medium.

The long CLS of the *ade* mutants was reminiscent of the CR effect, suggesting there could be some degree of overlap between the two. To test this idea, CLS of the WT and *ade4*Δ mutant was measured using the semi-quantitative spot growth assay (Figure 5A), and a quantitative colony forming unit assay that can detect more subtle changes in CLS (Figure 5B). Both assays showed there was no additive effect on CLS when combining the genetic factor (*ade4*Δ*)* and the environmental factor (CR), at least for the duration of the experiment (36 days). This was consistent with some overlap in function or involved pathways. To further test this possibility, we also examined the effect of deleting *ADE4* on the CLS of an autophagy mutant (*atg16*Δ). While the CR growth condition fully extended CLS of the *atg16*Δ mutant (Figure 2A), deleting *ADE4* from the *atg16*Δ mutant only resulted in a partial extension of CLS (Figure 4A). Therefore, one of the differences between CR and the *ade4*Δ mutant in CLS extension is a differential requirement for autophagy.

### Media effects on CLS

An earlier large-scale screen for long-lived yeast mutants did not uncover the *de novo* purine biosynthesis pathway genes [8]. We noticed that one of the differences between our study and the earlier study was the media composition used for the CLS assays. In general, the SC medium used in our study (Hopkins mix) is relatively rich in most amino acids compared to the SC medium used in the earlier study, which is described in Current Protocols in Molecular Biology [41], and abbreviated here as “CPMB” mix (Table S6). We compared the effects of each SC mix on the CLS of WT, *ade4*Δ, *atg16*Δ, and *fet3*Δ strains. As shown in Figure 5C, CLS of the WT strain was significantly longer in the CPMB media than in the Hopkins medium, even though glucose was 2% in both. As a result, the WT and *ade4*Δ lifespans were indistinguishable in the CPMB medium. Interestingly, the CPMB media did not extend the short CLS of the *atg16*Δ mutant (Figure 5C), even though reducing the glucose concentration in Hopkins medium fully extended its CLS (Figure 2A). Similar results were observed with several other autophagy mutants (data not shown), consistent with autophagy being required for mediating the effects of amino acid restriction on CLS. In contrast, the short CLS of the *fet3*Δ mutant, which was not extended by glucose CR (Figure 2B), was also not extended by the CPMB media (Figure 5C), making Fet3 important for mediating the effects of both glucose restriction and amino acid restriction on CLS.

### Cell-extrinsic effects on CLS

Considering the large effects of media composition on CLS, we next investigated whether any of the mutants isolated from the screen could influence longevity via cell-extrinsic factors that are secreted or released into the growth media. For example, secreted purine compounds such as adenine and hypoxanthine have previously been implicated in the regulation of meiosis within a sporulating yeast culture [42]. Additionally, we noticed during this study that expired medium from NR cultures would reverse the long CLS of CR-grown cells, and expired medium from CR cultures would extend CLS of NR-grown cells (D.L. Smith Jr., unpublished data). A similar finding was recently published by the Kaeberlein lab, who reported that acetic acid secreted into the medium during NR growth conditions correlated with the short lifespan, and that CR conditions prevented acetic acid secretion [43]. Reduced exposure to acetic acid in the CR cultures was specifically shown to extend CLS, therefore providing a possible mechanism for how CR extends CLS. Interestingly, other conditions that extend CLS such as high media osmolarity or deletion of *SCH9* have been proposed to make the cells more resistant to the acetic acid accumulation, rather than blocking organic acid production and secretion [43]. Taken together, these observations raised the question of whether any mutants isolated from our screen could affect CLS through a similar cell extrinsic mechanism.

**Figure 6:**
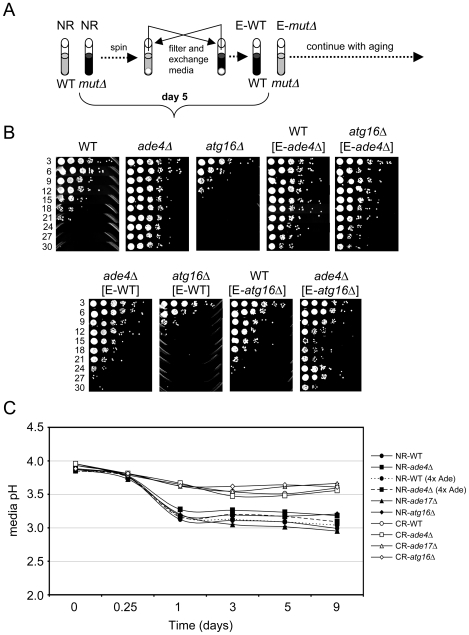
Cell-extrinsic effects of the *atg16*Δ and *ade4*Δ mutants on CLS. (A) Schematic diagram of a reciprocal media swap experiment. WT and mutant cell cultures were grown to day 5 in standard SC media containing 2% glucose (NR), at which point the cells were pelleted. The media was removed, filtered, and then exchanged such that the cell pellets received expired media derived from the mutant (E-*mut*Δ) or WT (E-WT) strains. The cultures were then allowed to age and the standard CLS assay continued. (B) CLS assay of the media swap experiment. WT, *ade4*Δ and *atg16*Δ strains without the swapped media are included as controls. (C) pH measurements of the SC growth media over time in NR and CR conditions. WT, *ade4*Δ, *ade17*Δ, and *atg16*Δ mutants were tested. A four-fold excess of adenine was added to the WT and *ade4*Δ strains under the NR condition where indicated.

To test for cell extrinsic effects we grew WT, *ade4*Δ, and *atg16*Δ strains in SC 2% glucose (NR) medium for 5 days into stationary phase. The cells were then pelleted and the expired medium was filtered and swapped in various combinations (Figure 6A). For example, the WT cells received expired medium from the *ade4*Δ or *atg16*Δ cells, and vice versa. The media-swapped cultures were then followed through a standard CLS assay (Figure 6B). Interestingly, the CLS of WT and *atg16*Δ cells was extended when incubated in expired medium from the long-lived *ade4*Δ cells. In the reciprocal swap, medium from the WT cells largely suppressed the long CLS of the *ade4*Δ mutant, but had no effect on the *atg16*Δ mutant. Expired medium from the short-lived *atg16*Δ mutant did not shorten CLS of the WT strain, but shortened CLS of the *ade4*Δ mutant (Figure 6B). The expired *atg16*Δ medium also tended to induce an adaptive regrowth effect, as shown in Figure 6B for the *ade4*Δ mutant, where nutrients released by dying cells in the stationary phase culture allow some of the remaining viable cells to regrow and populate the culture [44]. The *ade4*Δ and *atg16*Δ mutants therefore do alter the growth media in a way that can impact CLS.

**Figure 7:**
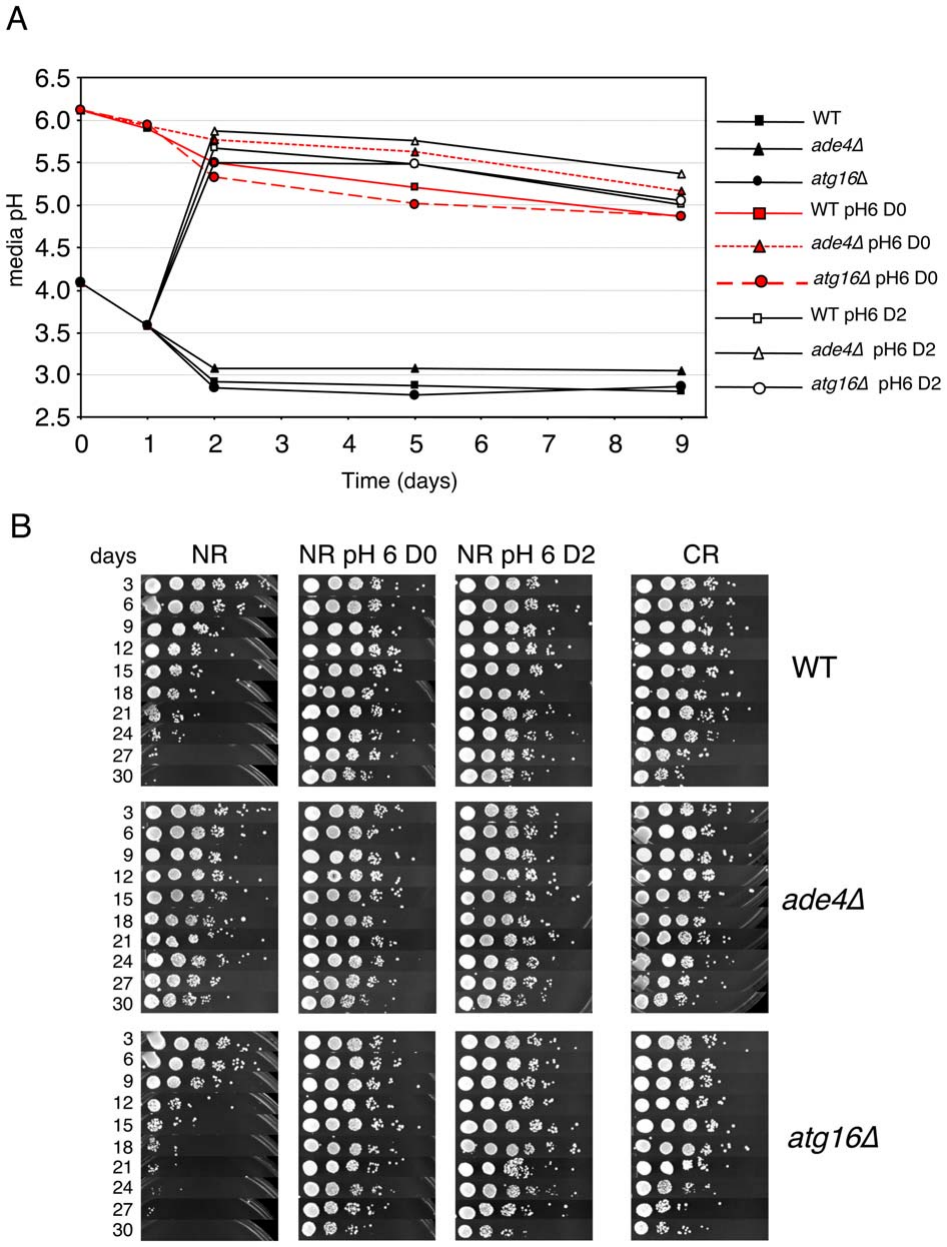
Effects of elevated pH on CLS. (A) Standard SC cultures (2% glucose) that were either not pH adjusted (started at the default pH of ∼4.0) or pre-adjusted to 6.0 were inoculated with the indicated strains. At day 2 (48 hours), the pH of a subset (open symbols) of the originally untreated cultures was adjusted to a pH of 6.0. For each culture, the pH was measured at the start of incubation (day 0), and then days 1, 2, 5, and 9. (B) CLS assays showing the effect of raising the SC medium pH to 6.0 at either at inoculation (D0), or after 2 days growth (D2).

The secretion of organic acids (including acetic acid) and CO_2_ into the growth medium during fermentation results in a reduction of pH. The toxicity of acetic acid on yeast cells requires a low pH [43]. Therefore, we next tested whether CLS of these mutants correlated with changes in media pH. WT, *ade4*Δ, *ade17*Δ, and *atg16*Δ strains were grown in SC medium containing 2% glucose (NR) or 0.5% glucose (CR), and the pH of the media was measured over time. As expected, the pH of NR medium for WT cells decreased from ∼3.9 to ∼3.15 during the first 24 hr of growth and then leveled off. For WT cells in CR medium, the pH still decreased, but only to ∼3.5 by day 5. Media from the short-lived *atg16*Δ and *ade17*Δ mutants had pH profiles across the time course that were similar to the long-lived *ade4*Δ mutant regardless of the starting glucose concentration, indicating that CLS did not correlate with overall pH of the media. However, the lack of a correlation between pH and CLS did not rule out the possibility that acetic acid could still be involved in the extrinsic CLS regulation, especially since the pH remained relatively low (<4.0) in each conditions. Furthermore, an acidic environment is not sufficient to chronologically age yeast cells in the absence of acetic acid [43]. If acetic acid was involved in the extrinsic CLS effects, then raising the medium pH close to neutral should suppress the relatively short CLS of the WT and *atg16*Δ strains. Indeed, raising the medium pH to 6.0 either at the time of inoculation (D0) or after two days of growth (D2) (Figure 7A), resulted in a dramatic extension of CLS for the WT and *atg16*Δ strains that was at least as strong as the *ade4*Δ mutant effect or the CR growth condition (Figure 7B).

**Figure 8:**
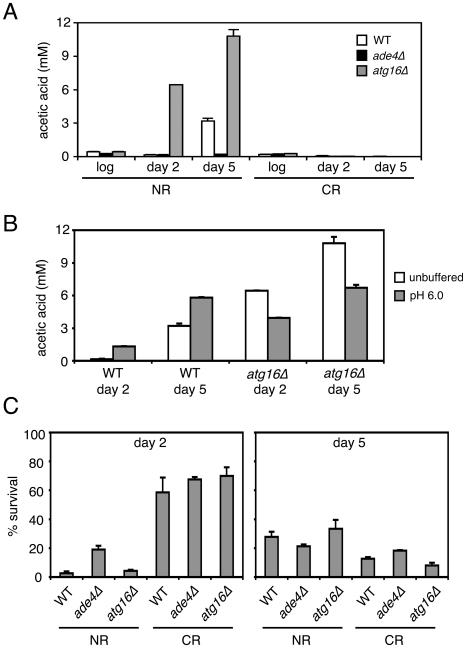
CLS mutants affect acetic acid secretion and resistance. (A) Acetic acid concentrations in the filtered media from cultures of WT, *ade4Δ*, and *atg16*Δ strains that were growing in 2% glucose (NR) or 0.5% glucose (CR) conditions. Measurements were taken from log phase cultures, or cultures grown for 2 and 5 days. (B) Acetic acid measurements from WT and *atg16*Δ strains grown in unbuffered SC, or SC with the pH adjusted to 6 at the time of inoculation. (C) Resistance of the three strains to a 200 minute exposure of 300 mM acetic acid was measured for cells that were grown for 2 or 5 days in SC media (NR or CR levels of glucose). Percent survival for the treated cells is normalized to the untreated cells, which would be 100%.

To determine whether the *ade4*Δ and *atg16Δ* mutants had any effect on acetic acid accumulation in the growth medium, the acetic acid concentration was measured from log phase, day 2, or day 5 cultures. As shown in Figure 8A, acetic acid accumulated to ∼3 mM in the WT culture on day 5. For the *atg16Δ* mutant, acetic acid accumulated earlier (day 2) and at a higher concentration by day 5 (∼11 mM), which was consistent with the short CLS of this mutant. In contrast, the long-lived *ade4Δ* mutant did not accumulate acetic acid at all compared to WT, which was very similar to the effect of CR on blocking acetic acid accumulation (Figure 8A). Therefore, the amount of acetic acid secreted into the medium for these two mutants was inversely correlated with their respective CLSs. Since the short CLS phenotype of the *atg16Δ* mutant was rescued by raising the pH to 6.0 (Figure 7), we were curious whether the higher pH was accompanied by a decrease in acetic acid concentration. The pH was again adjusted to 6.0 at the time of inoculation for WT and *atg16Δ* strains, and then acetic acid concentration measured at day 2 and day 5 (Figure 8B). Unexpectedly, the acetic acid concentration was elevated in the WT strain and reduced in the *atg16Δ* strain at both time points when the pH was adjusted to 6.0 at the time of inoculation (Figure 8B). Such variations in acetic acid accumulation apparently have no effect on CLS because the pH is too high to support the toxicity.

Since a long-lived *sch9*Δ mutant was previously shown to make yeast cells more resistant to acetic acid [43], we tested whether the *ade4Δ* and *atg16Δ* mutations affected cell survival when cultures grown for 2 or 5 days were challenged with 300 mM acetic acid for 200 minutes (Figure 8B). In the day 2 cultures, the *ade4Δ* mutant was significantly more resistant to acetic acid than the WT strain, again consistent with the long CLS of this mutant. However, resistance of the *atg16Δ* mutant was indistinguishable from WT. The CR condition made all three strains highly resistant to the acetic acid treatment. In the day 5 NR cultures (the time of the media swaps in Figure 6), there were no significant differences in the acetic acid resistance between the three strains, and surprisingly, the CR growth condition no longer made the cells more resistant. Resistance to acetic acid could potentially play a role in CLS extension for the *ade4Δ* mutant, which would be consistent with its ability to survive in the pooled mutant culture used for the screen, where many mutants would secrete acetic acid. In contrast, the short CLS of the *atg16Δ* mutant may not be due to acetic acid hypersensitivity. These results suggest that secreted acetic acid can commonly impact CLS through a cell extrinsic mechanism that is dependent on media pH.

## Discussion

A microarray-based screen for short- and long-lived mutants from the YKO collection led to the identification of several pathways that regulate CLS, including autophagy and the *de novo* purine biosynthesis pathway. An earlier screen for chronologically long-lived deletion mutants revealed that reduced TOR signaling extends CLS [8]. The strongest TOR-related mutant from that screen was a *gln3* deletion. In our screen, the *gln3*Δ mutant just missed the conservative selection criteria because its day 33 abundance ratios fell outside the top 500 (781 for DNTAG and 738 for UPTAG). However, its day 21 ratios were much higher than 1.0, consistent with the long CLS that was observed when tested directly (Figure 4). A direct test of *gln3*Δ and *tor1*Δ mutants also confirmed that TOR signaling controlled CLS in haploid yeast and growth media used in our study. A total of 117 potential short-lived mutants were isolated from the screen, with 13 of the 16 individually retested mutants confirmed to have a short CLS. Similarly, a total of 40 potential long-lived mutants were isolated, with 12 of the 39 retested mutants confirmed to have an extended CLS. From all the mutants tested individually for various reasons as part of this study, 69 short-lived and 57 long-lived mutants were found to affect CLS and are listed in Table S3 and Table S4, respectively.

### Autophagy is required for chronological longevity in yeast

Autophagy is a multi-step process in which a portion of the cytoplasm is sequestered into a *de novo*-formed double membrane vesicle called the autophagosome. These vesicles fuse with a lysosome (the vacuole in yeast) and release the inner single-membrane vesicle called the autophagic body. Any sequestered organelle or other cellular matter in the autophagic body is degraded and recycled into amino acids, fatty acids, sugars, etc. [45]. This process is especially important during times of stress when cellular components can become damaged and aggregate, or when nutrients are depleted. Chronological aging of yeast cells is characterized by the ability to survive during extended incubation in starvation phase, making the ability to recycle resources critical. The identification of multiple deletion mutants in the autophagy pathway that shorten CLS therefore makes sense, not only because of the need to regenerate cellular components, but to potentially eliminate damaged proteins that arise as the cells age. Our results are consistent with results in *Drosophila* where mutation of the *ATG7* gene shortens lifespan [46], and a more recent study in yeast showing that *atg1*Δ and *atg7*Δ mutants have a short CLS in synthetic growth medium [47]. The *atg7*Δ mutant was one of the autophagy mutants also isolated from our screen. Surprisingly, most autophagy gene deletion mutants have a normal RLS in rich YPD medium [48]. Similarly, the *atg16*Δ mutant had a normal RLS when we tested it in SC medium (Figure S1), making it a CLS-specific longevity factor.

Disruption of autophagy in *C. elegans* prevents the extension of lifespan caused by a *daf-2* mutation or dietary restriction [21], [22], [49]. Deleting *ATG15*, but not the other autophagy genes, blocks CR-mediated RLS extension in yeast [48]. *ATG15* was not isolated from our screen, and hence not tested for CLS, but every other autophagy mutant we tested responded to CR with CLS extension (Figure 2A). Interestingly, we found that deleting *ATG16*, *ATG2*, or *ATG6* (*VPS30*) prevented CLS extension induced by the CPMB variety of SC media used in the Powers et al. screen, which has a normal 2% glucose level but generally has lower concentrations of amino acids compared to the Hopkins mix (Figure 5C). This result is consistent with the strong stimulation of autophagy triggered by nitrogen limitation or amino acid depletion [50], [51]. Indeed, maintenance of amino acid homeostasis via the general amino acid control system is important for proper CLS [47]. Furthermore, the long CLS of a *tor1*Δ mutant requires the autophagy gene *ATG16* (data not shown). Similarly, autophagy was recently shown to be required for the extension of CLS induced by low concentrations of rapamycin [52], an inhibitor of the TOR signaling pathway. Future studies on the links between autophagy, amino acid depletion, and lifespan extension are clearly warranted.

### Iron metabolism and caloric restriction

Two proteins (Fet3 and Nfu1) involved in iron homeostasis/metabolism were isolated as mutants whose CLS was not extended by the CR growth condition. Iron accumulates to high levels in the vacuole of yeast cells where it can be accessed during times of need, such as low iron growth conditions. Another key time of iron release from the vacuole is during the diauxic shift when the balance of iron is shifted to the mitochondria, where it is needed for mitochondrial biogenesis. The iron is incorporated into iron/sulfur complexes within multiple mitochondrial proteins, including aconitase and components of the electron transport chain. A defect in iron homeostasis could affect mitochondrial processes. One of the phenotypes observed during chronological aging is an accumulation of intracellular iron. Much of this iron is likely tied up in lipofuscin, an insoluble aggregate of proteins and lipid that is high in iron and accumulates in aging cells. Interestingly, CR reduces this accumulation of lipofuscin and iron [53]. The reduction in iron could contribute to the corresponding reduction in reactive oxygen species because a major source of reactive oxygen species is generated via iron through the Fenton reaction. It is not clear why a *fet3*Δ mutant would block the CR effect, but perhaps the iron oxidase activity of Fet3 has an additional function in iron homeostasis beyond its role in high affinity transport. Interestingly, a recent report showed that *FET3* is one of several iron related genes that are up-regulated in response to increasing strength of CR [54]. *FET3* was also required for the extension of CLS induced by the low amino acid CPMB medium (Figure 5C), pointing to iron and possibly mitochrondrial function being important for both glucose and amino acid restriction effects on CLS.

### The *de novo* purine biosynthesis pathway and longevity

The *de novo* purine biosynthesis pathway is familiar to yeast researchers because the AIR intermediate that accumulates in *ade2* mutants takes on a red pigmentation when it is oxidized and concentrated in the vacuole of respiring cells. Multiple genetic assays have taken advantage of this visual phenotype [55], [56]. Limiting the amount of adenine in the growth medium promotes development of the red color by increasing flux through the pathway. The 30 mg/L of adenine in Hopkins mix SC is limiting in this context. Excess adenine suppresses the red color by reducing flux through the pathway, thus reducing AIR formation. Excess (4X) adenine also suppresses the long CLS of the *ade2*Δ, *ade3*Δ, and *ade4*Δ mutants, but had no effect on CLS of the WT strain. One possible mechanism for a block in this pathway to regulate CLS is that reduced production of AMP and/or IMP leads to lifespan extension. Consistent with this idea, deletion of the adenylate kinase 1 gene *ADK1*, which leads to a large increase in cellular AMP concentration [57], also shortens CLS (Table S3). AMP is an allosteric effector of multiple enzymes in metabolism, including phosphofructokinase (PFK), a key regulatory step in the glycolytic pathway who's activity is enhanced by AMP binding. CR has been shown to reduce PFK activity in mouse liver [58]. Lower AMP levels could mimic CR by reducing glycolytic flux. This model also fits the extended CLS of the *fcy2*Δ mutant, which would also reduce AMP production by blocking the import of extracellular adenine. The compensatory increase in AMP production by the *de novo* purine synthesis pathway would partially suppress the effect, resulting in the more modest increase in lifespan for this mutant compared to the *ade4*Δ mutant. Since the *de novo* purine biosynthesis pathway and Fcy2-mediated transport of guanine also regulate GMP production (and subsequently GTP/GDP levels, reduced GMP levels could also contribute to the lifespan extension via effects on the Ras/cAMP/PKA pathway, as inhibition of Ras2 results in extension of CLS [59]. Consistent with this possibility, we have found that deletion of *BCY1*, which constitutively activates PKA, shortens CLS (Table S3).

A second possible mechanism for the *de novo* purine biosynthesis pathway to regulate CLS is through the control of AICAR concentration. Severe accumulation of AICAR induced by *ADE4* over-expression in an *ade16 ade17* double mutant causes synthetic lethality [40]. The less severe accumulation predicted for an *ade17*Δ mutant is not lethal, but instead leads to a short CLS (Figure 3C). However, any putative negative effect of AICAR accumulation from a defect in this step of the pathway is overcome, in terms of CLS, by a double deletion of *ADE16* and *ADE17*. This double mutant behaves like any other deletion mutant in the *de novo* pathway (long-lived), suggesting that effects on IMP/AMP production or other unknown mechanisms are dominant to the AICAR effect. If AICAR does have a negative effect on CLS, then it is modest and opposite of that observed in higher eukaryotes. In metazoans, AICAR acts as an agonist of AMP-activated protein kinase (AMPK) [60], an enzyme that functions in mediating some aspects of longevity in *C. elegans* [61], [62]. The yeast paralog of AMPK, Snf1, is not activated by AMP or AICAR [63]. Furthermore, the *snf1*Δ mutant was found to have a short CLS (Table S2), a phenotype that is likely due to the roles of Snf1 in promoting respiration and autophagy [64], [65]. Given the complex nature of purine biosynthesis regulation and its links to the regulation of other metabolic pathways, including amino acid biosynthesis, other mechanisms leading to lifespan extension are certainly possible. For example, secreted adenine-related compounds could contribute to the cell-extrinsic effects of the *ade* mutants on CLS. In fact, the temporal secretion of various purines into the media and their subsequent uptake and utilization is a key signal that synchronizes the sporulation process between cells in a dense culture [42].

### Acetic acid and the regulation of CLS

Acetic acid accumulates to low millimolar concentrations in stationary phase yeast cultures that are grown in SC medium with 2% glucose (NR). Exposure to this acetic acid, coupled with the acidic environment of the expired medium contributes to chronological aging [43]. CR growth conditions block the acetic acid accumulation, and long-lived mutants such as *sch9*Δ and *ras2*Δ tend to be resistant to acetic acid toxicity, suggesting that resistance to acetic acid may be a general property of chronologically long-lived yeast cells [43]. We found that the long-lived *ade4*Δ mutant blocked acetic acid accumulation in the growth medium as effectively as CR, while the short-lived *atg16*Δ mutant accumulated significantly higher concentrations of acetic acid than did the WT strain (Figure 8A). In addition to greatly reducing acetic acid levels (Figure 8A), we found that CR makes all three strains more resistant to acetic acid when the exposure occurs after 2 days growth, but is no longer effective with 5-day cultures (Figure 8B). While the *ade4*Δ mutant was moderately resistant to acetic acid at day 2 when compared to WT, by day 5 there was very little difference in sensitivity between the two mutants and WT. This is an important point, because the expired media swaps between the WT, *ade4*Δ, and *atg16*Δ strains were performed with 5-day old cultures. Perhaps chronologically aged yeast cells are simply programmed to be more resistant to acetic acid as a defense against this by-product of fermentation. These were short-term acetic acid exposures (200 minutes), so it is possible that prolonged exposure of the day 5 cultures, or lack of exposure for the *ade4*Δ expired media, could still affect CLS. This would also correlate well with the extension of CLS induced by raising the pH to 6.0 (Figure 7B), which would neutralize the toxicity of acetic acid. The *ade4*Δ mutation therefore both suppresses acetic acid accumulation and promotes acetic acid resistance, a phenotypic combination also induced by the CR growth condition.

It remains unclear why a defect in autophagy (*atg16*Δ) results in hyper-accumulation of acetic acid, while a block in *de novo* purine biosynthesis prevents acetic acid accumulation. An important function of autophagy is the turnover of organelles, including mitochondria. In mice deficient for Atg7, mitochondrial dysfunction has been observed that is accompanied by elevated reactive oxygen species [66]. Perhaps a defect in mitochondrial function would promote fermentation during NR conditions by preventing the yeast cells from fully transitioning from fermentation to respiration at the typical diauxic shift, and thus favoring acetic acid production. This would also account for the large number of mitochondria-related mutants that were isolated from the screen as being short-lived. Given the similarities of the *ade4*Δ CLS phenotype to CR, it is possible that the *ade4*Δ mutant could also enhance a shift from fermentation toward respiration, which could reduce acetic acid production. For the various mutants isolated from the screen, it will therefore be interesting to further compare the relative CLS contributions of their actual cellular defects with their acetic acid secretion and toxicity profiles. Specific combinations of intracellular and extracellular effects are likely going to be CLS determinants.

### Efficacy of large-scale screens for chronological aging factors

The microarray-based genetic screen performed in this study was successful in identifying several novel longevity genes, but its quantitative ability to predict long-lived mutants based on the abundance ratios from the arrays was modest. Similar difficulties were previously observed using the YKO collection in a different type of longevity screen, in which each mutant was individually grown in a 96-well plate, and ability to re-grow was tested over time. In that screen, only 5 of 90 predicted long-lived mutants (5.6%) were confirmed when retested [8], [67]. In our case, 12 of the 39 candidate mutants (30.8%) were confirmed as long-lived when retested (Table 1). Not surprisingly then, only 4 of the 12 confirmed long-lived mutants isolated from our screen (*LCL1*, *DCW1*, *LCL2*, and *MUM2*) were ranked in the top 1000 long-lived candidates from the earlier Powers et al. CLS screen. These results are likely indicative of inherent variability in large-scale screens for long CLS, as well as subtle differences in the growth conditions. Large-scale screening for short-lived mutants is much more efficient, which is reflected in the fact that 68 of the 117 short-lived candidates from our screen (58.1%) are also in the bottom 1000 short-lived candidates from the Powers et al. screen (Table S2). Having multiple screening approaches is advantageous, as mutants not detected by one method may be detected by another.

There are several possible reasons for the variability associated the microarray-based longevity screen, especially for long-lived mutants. One possibility is the adaptive regrowth phenomenon, in which a subpopulation of cells in an aging stationary phase culture adapts to utilize the nutrients released by dead cells to re-grow and populate the culture [44]. If a mutant underwent gasping during aging of the pooled collection, then it would register an artificially high abundance ratio, and fail to be long-lived when individually retested. Another possibility that would be unique to the mixed population approach is the introduction of competition between the strains, where mutants with improved overall fitness could have an advantage that is lost when they are retested individually. In a related scenario, certain mutants in the mixed population are likely highly resistant or overly sensitive to changes in medium composition (such as acetic accumulation) that occurred as the cultures were aging. Certain mutants could directly influence the medium composition, thus altering the lifespan of the highly sensitive mutants in the process. A good example is the *ade4*Δ mutant, whose expired SC medium extended the lifespan of the WT and *atg16*Δ strains (Figure 6), possibly through the reduction of acetic acid accumulation (Figure 8A). In applying the microarray/barcode approach to other aging or age-related problems, it is likely that the amount of variability would be more limited with the addition of duplicate or triplicate screens. However, even with the inherent variability, this microarray screen successfully identified several novel longevity regulators that will be the subject of future studies.

## Materials and Methods

### Yeast strains and media

Yeast strains used in this study were isogenic to the haploid strain BY4741 (*MAT* ***a*** *his3*Δ*1 leu2*Δ*0 met15*Δ*0 ura3*Δ*0*), and were obtained from the yeast gene knockout collection [19]. The *ade16*Δ *ade17*Δ mutant strain (Y1093) was kindly provided by Bertrand Daignon-Fornier [68]. Most *in vivo* assays were performed in synthetic complete (SC) medium following the recipe provided in the Cold Spring Harbor Yeast Genetics Course Manual [69], and sold by QBioGene as “Hopkins mix”. The alternative SC medium is derived from Current Protocols in Molecular Biology [41], which we refer to as “CPMB” mix. Chemical compositions of the various SC media types are listed in Table S6. Glucose was added to the SC media to a final concentration of either 0.5% (CR-Calorie Restricted) or 2% (NR-Non Restricted). Where indicated, the Hopkins mix SC medium was buffered to pH 6.0 with a citrate phosphate buffer (6.42 mM Na_2_HPO_4_ and 1.79 mM citric acid, final concentration), as previously described [43]. For buffering the medium at day 2, a 10× concentrate of the citrate phosphate buffer was added to SC. For pH measurements of expired media, small aliquots were removed from the cultures and then discarded to prevent contamination of the long-term culture.

### Genetic screen for longevity and TAG–microarray analysis

To begin the screen, 1 ml (15 OD_600_ units) of the pooled haploid knockout collection was inoculated into 200 ml SC medium containing either 2% glucose (NR) or 0.5% glucose (CR). The next day (day 0), aliquots of 100 µl were transferred into 10 ml of fresh SC-NR and SC-CR media, respectively. Twenty such cultures were inoculated for each glucose concentration and allowed to age at 30°C in the roller drum to provide aeration [16]. Starting with day 1 (D_1_), 100 µl of each culture was plated onto YPD plates every 3 days to allow viable cells in the population to re-grow. These YPD plates were incubated at 30°C for 2 days and the cell lawns harvested by scraping and pooled together, then washed with ice cold water and stored at −80°C. Once the time course was completed (day 33), genomic DNA was isolated from the cell pellets [41].

The UP- and DNTAGs were labeled with Cy5 (day 1) or Cy3 (days 9, 21, and 33) by PCR amplification of genomic DNA using primer pairs U1/U2 and D1/D2, respectively, as previously described [70]. The Cy5-labeled UP- and DNTAGs from day 1 were then co-hybridized with the Cy3-labeled UP- and DNTAGSs on custom-designed “Hopkins TAG-arrays” from Agilent Technologies (AMADID 011443) as previously described [70]. Fluorescence signal intensities were measured by scanning the arrays with a Genepix 4000B instrument coupled with GenePix Pro software. The signal intensity ratios were then calculated for days 9, 21, and 33 compared to day 1 as the control using Microsoft Excel. The signal ratios for all essential genes on the array were averaged and considered the background. Any non-essential genes with up- or down-tag ratios lower than this background average were eliminated from the analysis, thus ensuring that only genes with signals from both tags were included (2715 genes, which included most of those in the DNTAG list in Table S1). Box plots of the ratios in Figure 1 were assembled from the 3478 genes in the UPTAG list (Table S1) using R Software. Mutants with similar average NR and CR log ratios were identified by applying two criteria to their values at every time point: (1) ratios were within 10% of each other and (2) the null hypothesis that were the same according to a t-test. In the case of (1), we calculated the fractional difference between the average NR and CR log ratios (i.e., difference between these values divided by their average). The absolute value of the fractional difference was required to be less than 0.1. We then applied a t-test to the NR and CR log ratios and required their p-value to be less than 0.05 (i.e., their means are not significantly different).

### Chronological life span assays

Quantitative (colony forming unit) and semi-quantitative (10-fold serial dilution spot-test) chronological life span (CLS) assays were performed as previously described [16]. For the media swap experiments, the 10 ml cultures were grown for 5 days. The cultures were then pelleted in a swinging bucket rotor (2500 RPM) at room temperature in an Eppendorf 5810R tabletop centrifuge. The supernatants were removed and passed through a 0.2 micron syringe filter prior to the swap.

### Acetic acid measurements and treatments

For the measurement of acetic acid concentration in growth media, cells were grown in the appropriate SC medium (10 ml in culture tubes) to the indicated time points. Log phase cells (OD_600_ of 0.8) and cells grown to day 2 and day 5 were pelleted by centrifugation, and the clarified media was passed through a 0.2 micron syringe filter. The filtrate was used for measuring the acetic acid concentration using an Acetic Acid Kit (R-Biopharm AG, Darmstadt, Germany), following the manufacturer's directions. Three biological replicas were assayed for each condition to provide mean millimolar concentrations and standard deviations. To determine sensitivity/resistance of the mutant strains to exogenously added acetic acid, cultures were challenged for 200 minutes with 300 mM acetic acid either at day 2 or day 5 of the CLS assay. Cells were diluted in water and then spread onto YPD plates to allow viable cells to grow into colonies, which were then counted. The percent survival was calculated by dividing the colony forming units (CFU) of the treated samples by the untreated samples. Three biological replicates were tested for each condition.

## Supporting Information

Figure S1 Replicative lifespan (RLS) measurements of WT (BY4741), *atg16Δ*, and *ade4Δ* strains. For each strain, a total of 70 mother cells were analyzed on SC media containing 2% glucose. Mean RLS values were as follows: WT (25.4), *atg16Δ* (25.2), and *ade4Δ* (24.9).
(0.16 MB PDF)

Table S1 Ranks of mutants for each particular time point and glucose condition.
(3.20 MB XLS)

Table S2 Predictions of putative short-lived mutants.
(0.06 MB XLS)

Table S3 List of tested mutants with shortened CLS.
(0.03 MB XLS)

Table S4 List of tested mutants with extended CLS.
(0.03 MB XLS)

Table S5 List of tested mutants with no effect on lifespan.
(0.03 MB XLS)

Table S6 Chemical composition of various SC media recipes.
(0.02 MB XLS)
