# Supplementary material for: A Microarray-Based Genetic Screen for Yeast Chronological Aging Factors
Source: PLoS Genet. 2010 Apr 22;6(4):e1000921. doi: 10.1371/journal.pgen.1000921 (PMC2858703; doi:10.1371/journal.pgen.1000921)
Supplement: Figure S1 — Replicative lifespan (RLS) measurements of WT (BY4741), atg16Δ, and ade4Δ strains. For each strain, a total of 70 mother cells were analyzed on SC media containing 2% glucose. Mean RLS values were as follows: WT (25.4), atg16Δ (25.2), and ade4Δ (24.9). (0.16 MB PDF) [file pgen.1000921.s001.pdf]

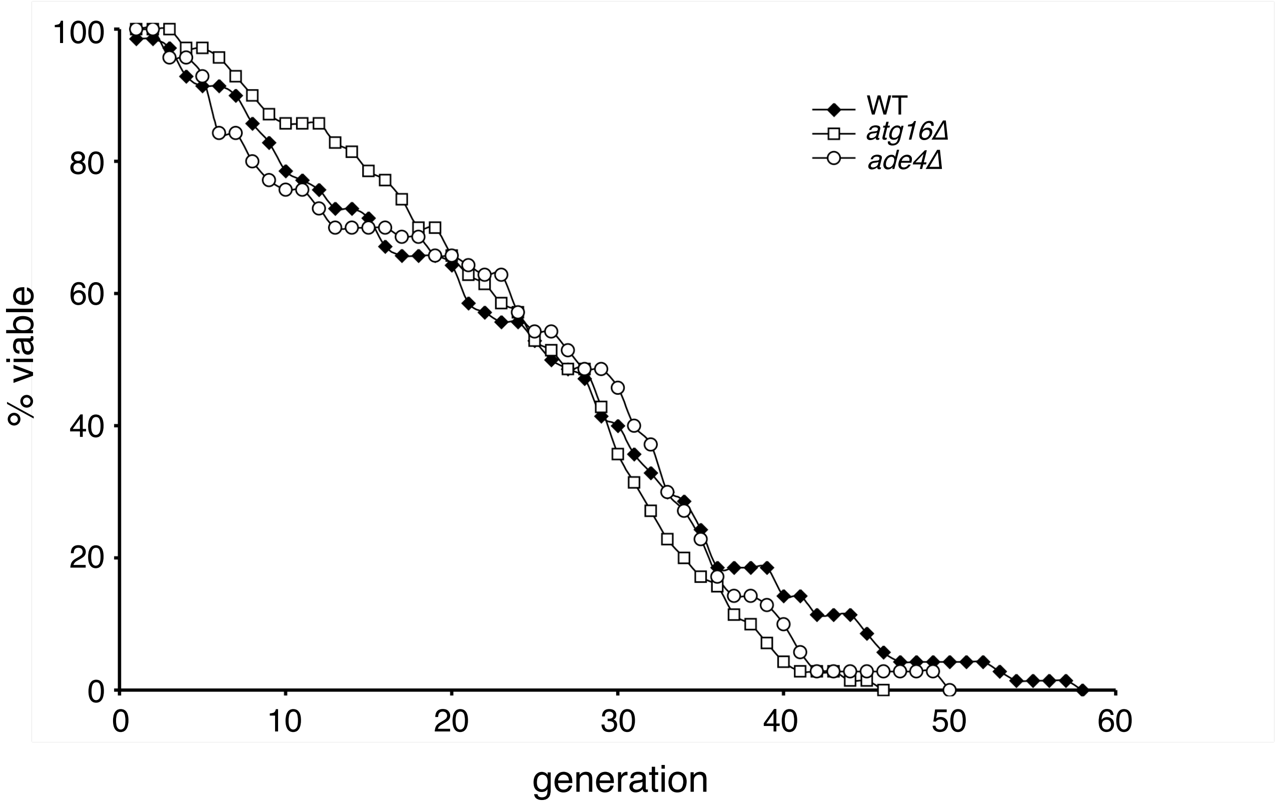

Figure S1. Replicative lifespan (RLS) measurements of WT (BY4741), *atg16*Δ*,* and *ade4*Δ strains. For each strain, a total of 70 mother cells were analyzed on SC media containing 2% glucose. Mean RLS values were as follows: WT (25.4), *atg16*Δ (25.2), and *ade4*Δ (24.9).
